# Sustainable Valorisation of Coffee Waste as a Protein Source, Mycelium-Based Packaging Material and Renewable Energy Pellet

**DOI:** 10.3390/molecules29214983

**Published:** 2024-10-22

**Authors:** Anca Becze, Dorina Simedru, Daniel-Gabriel Barta, Lacrimioara Senila, Cerasel Varaticeanu, Tudor Blaga

**Affiliations:** 1 National Institute for Research and Development of Optoelectronics INOE 2000, Research Institute for Analytical Instrumentation, 67 Donath Str., 400293 Cluj-Napoca, Romania; dorina.simedru@icia.ro (D.S.); lacri.senila@icia.ro (L.S.); cerasel.varaticeanu@icia.ro (C.V.); tudor.blaga@icia.ro (T.B.); 2 B&G Family Innovation SRL, Street Tăutului 242B, 407280 Cluj-Napoca, Romania; barta.gabriel90@gmail.com

**Keywords:** spent coffee grounds, alternative protein, mycelium-based packaging, renewable energy, green extraction

## Abstract

This study investigates the valorization of spent coffee grounds (SCGs) through protein extraction and their application in mycelium-based packaging and renewable energy pellets. Three extraction methods—mechanical stirring, ultrasound-assisted, and CO_2_-assisted extraction—were applied to SCGs. CO_2_-assisted extraction yielded the highest protein content at 34.24%, followed by mechanical stirring (31.46%) and ultrasound-assisted extraction (28.51%). The total polyphenol content and antioxidant capacity were also highest in the CO_2_ extracts, suggesting that this method preserves bioactive compounds most effectively. After protein extraction, SCGs were tested as a component in mycelium-based packaging, with results showing an apparent density of 0.551 g/cm^3^ and compression resistance of 3.354 MPa, indicating its suitability for structural applications. The energy value of SCGs remained high, with a calorific value of 19,887 J/g DW, slightly decreasing after extraction but still sufficient for renewable energy production. These findings highlight the potential of SCGs as a multi-functional resource, contributing to sustainable solutions across various industries.

## 1. Introduction

In recent decades, the global consumption of coffee has surged, fuelled by evolving consumer preferences and the rise of specialty coffee culture [1,2]. As a result, the production of coffee waste has increased substantially, with millions of tons generated annually [3,4]. This waste, if not managed effectively, poses a significant environmental challenge. Typically disposed of in landfills, coffee waste contributes to greenhouse gas emissions, soil contamination, and the depletion of natural resources [5]. This growing problem emphasizes the need for innovative waste management strategies that mitigate its environmental impact.

Coffee consumption has experienced steady growth in recent decades, particularly in Europe, which holds the largest share of the global coffee market [6]. In 2022, Europe accounted for 31% of worldwide coffee consumption, translating to 55,000 bags of coffee annually [7,8]. Germany stands as the largest importer and trade hub for green coffee in Europe, re-exporting 168,000 tonnes of green coffee to neighboring countries such as Poland (63,300 tonnes), France (16,500 tonnes), and the Czech Republic (14,600 tonnes). Belgium follows closely, with 97,700 tonnes of green coffee re-exported annually, mainly to the Netherlands and France [7,8].

The European market for coffee is dominated by green coffee imports, which constitute 95% of all coffee imports because of several factors, including longer shelf life and favorable import tariffs [7,8,9]. In contrast, roasted coffee faces higher duties—7.5% for roasted coffee and 9% for decaffeinated roasted coffee—which encourages European companies to import green coffee for local processing. As a result, spent coffee grounds, coffee chaff, and other coffee waste materials accumulate in large quantities across the region [7,8,9,10].

The environmental challenge posed by this coffee waste is significant. Disposing of coffee waste in landfills contributes to greenhouse gas emissions and soil contamination, exacerbating the environmental impact of coffee production [11,12]. The sheer scale of coffee waste necessitates innovative and sustainable approaches to waste management.

The composition of coffee waste, which primarily consists of spent coffee grounds, coffee pulp, and coffee chaff, presents both challenges and opportunities for sustainable management [13,14]. Rich in organic compounds such as caffeine, polyphenols, proteins, and lipids, coffee waste can serve as a valuable raw material in numerous processes [3,15,16,17,18]. However, if left unutilized, these compounds can contribute to environmental degradation through leaching, methane emission, and other harmful effects [19].

In response to an increase in the accumulation of coffee waste, researchers and innovators are actively exploring sustainable methods for repurposing these byproducts.

Spent coffee grounds (SCGs), a byproduct of coffee brewing, offer promising potential for sustainable applications across various industries [20,21,22,23,24]. To determine their suitability for different uses, a range of physical, chemical, and biological tests are performed. The moisture content of SCGs affects their handling, storage, and susceptibility to spoilage, while the ash content provides insight into the inorganic mineral matter left after combustion, which is relevant for both biofuel production and soil amendment [25,26,27]. The pH level of SCGs is also important, as it influences its applicability in processes such as composting and environmental remediation [28]. Another key parameter is the carbon-to-nitrogen (C/N) ratio, which is essential for optimizing SCGs in composting or as a soil enhancer [21,24]. Particle size distribution and caffeine content are crucial for applications in food additives or pharmaceuticals, where the physical texture and residual caffeine levels impact product quality [20]. On the biological side, microbial activity within SCGs can be harnessed for composting or bioremediation, while their nutrient content, including nitrogen, phosphorus, and potassium, makes them a valuable component for soil enrichment [28,29]. Moreover, SCGs have demonstrated antimicrobial properties, making them potentially useful for inhibiting harmful microorganisms [30,31]. These comprehensive assessments enable researchers and industry experts to explore specific applications for SCGs, such as composting, soil amendments, biofuel production, food and beverage additives, and environmental remediation. Studies have shown that SCGs can absorb pollutants from water and soil, enhance soil structure, provide essential nutrients, and even serve as a sustainable energy source when converted into biofuels [13,32]. By unlocking the potential of SCGs, industries can reduce waste, create valuable products, and contribute to a more sustainable circular economy.

Mycelium, the root-like structure of fungi, can grow on organic substrates such as spent coffee grounds to form a durable, biodegradable material. Acting as a natural adhesive, mycelium binds particles into a solid composite that can be customized for flexibility, rigidity, or insulation. It is energy-efficient to produce, growing at ambient temperatures without chemicals, and fully biodegradable, supporting a circular economy. Mycelium packaging is being explored as an eco-friendly alternative to materials such as polystyrene, offering strength and insulation for various packaging applications [33]. This study aims to explore the application of spent coffee grounds as a sustainable protein source. SCGs contain an estimated protein content of 10–15%, depending on the coffee variety and extraction method, making them a promising alternative in the growing market for sustainable foods. This research focuses on developing a low-impact extraction technology that employs green methods, minimizing environmental impact. The protein content of SCGs can vary depending on the coffee variety, extraction method, and roasting intensity. For example, lighter roasts may preserve more proteins, while the extraction method (e.g., mechanical stirring, ultrasound-assisted, or CO_2_-assisted) influences the efficiency of protein recovery. Additionally, the study seeks to maximize the utilization of SCGs by repurposing the residual solid mass left after protein extraction.

## 2. Results and Discussion

### 2.1. SCG Characterization

The results obtained after the characterization of SCGs are presented in Table 1. The values provide a detailed analysis of key components present in spent coffee grounds, including protein, lipids, ash, total polyphenols, antioxidant capacity, and aflatoxin content. These data show that SCGs contain an average protein content of 15.77% DW, which aligns with this study’s aim to explore SCGs as a sustainable protein source. Additionally, the lipid content is measured at 15.25% DW, indicating a relatively balanced macronutrient profile that could be further utilized in various applications. The total polyphenol content, 1112.92 mg GAE/kg, highlights the rich antioxidant potential of SCGs, with an antioxidant capacity of 2.60 mg vitamin C/g. These values reinforce the potential of SCGs not only as a protein source but also as a functional ingredient rich in bioactive compounds. Zengin et al. (2020) reported significantly higher levels of polyphenols in spent coffee grounds (56.86–93.55 mg GAE/kg). This difference could be attributable to several factors, including variations in extraction methods, the type of coffee used, or environmental conditions such as brewing time and temperature [34]. Additionally, the storage time of SCGs before extraction may have influenced the polyphenol degradation, further contributing to the lower concentrations observed in our results [34]. The ash content of 6.89% DW suggests a moderate mineral presence, which could be valuable depending on the application. The results are similar to that of Franca et al. (2020) for proteins, who reported a protein content of 10–17%, but a lipid content is approximately 5% lower, and the ash content is 1% higher. This could be due to the analysis methods applied but also to the coffee variety and processing parameters [20].

To determine the statistical relevance of these findings, one-way ANOVA was performed across multiple samples to assess the variation within and between groups for key components such as proteins, lipids, and polyphenols. The results showed no significant differences (*p* > 0.05) in protein and lipid contents across samples, suggesting consistency in SCG composition. However, significant differences (*p* < 0.05) were found in the total polyphenol content, which could be attributable to batch variability or differences in coffee processing methods.

Regarding aflatoxin contamination, Aflatoxin B1 was detected in some samples at an average level of 0.25 µg/kg, while Aflatoxin B2, G1, and G2 were either not detected or present in minimal quantities, remaining well below regulatory safety limits [35]. This suggests that SCGs are generally safe for further use but that Aflatoxin B1 contamination should be monitored in certain cases.

The low standard deviations for components such as lipids (SD = 0.24) and ash (SD = 0.02) indicate high consistency across samples. These results, combined with ANOVA, demonstrate the statistical reliability of these data, reinforcing the potential of SCGs for sustainable food production, mycelium-based packaging, and renewable energy pellets.

Table 2 presents the concentrations of polycyclic aromatic hydrocarbons (PAHs) in spent coffee grounds (SCGs), with values expressed as mean ± standard deviation (SD). A one-way analysis of variance (ANOVA) was conducted to assess the variability in PAH concentrations across different SCG samples.

Among the detectable PAHs, phenanthrene had the highest concentration (0.0066 ± 0.0032 µg/kg), followed by fluoranthene (0.0031 ± 0.018 µg/kg) and naphthalene (0.0021 ± 0.014 µg/kg). These compounds exhibited high standard deviations, indicating significant variability, with some samples showing detectable levels and others not. This was further confirmed by ANOVA results, which showed significant differences (*p* < 0.05) in the concentrations of these PAHs between different SCG samples, reflecting their inconsistent presence across batches. On the other hand, compounds such as anthracene (0.0003 ± 0.00007 µg/kg), pyrene (0.0013 ± 0.0009 µg/kg), benz[a]anthracene (0.0004 ± 0.0005 µg/kg), chrysene (0.0002 ± 0.0004 µg/kg), benzo[ghi]perylene (0.0001 ± 0.0003 µg/kg), and indeno[1,2,3-cd]pyrene (0.0001 ± 0.0002 µg/kg) were detected at consistently low concentrations with smaller standard deviations, indicating a more uniform presence across the samples. Several PAHs, including acenaphthene, fluorene, benzo[b]fluoranthene, benzo[k]fluoranthene, benzo[a]pyrene, and dibenzo[a,h]anthracene, were below the limit of quantification (LQ), indicating their negligible presence in SCG. The total PAH concentration was 0.0141 ± 0.0052 µg/kg. The variability in PAH concentrations, especially for those with high SDs, points to inconsistent contamination across the samples. We did not find any current data on PAH levels in SCGs to be able to compare the results.

The results in Table 2 show that the concentrations of polycyclic aromatic hydrocarbons (PAHs) in spent coffee grounds (SCGs) are below the established regulatory limits [36,37]. In Europe, there is growing concern about PAHs, a group of contaminants that can originate from the drying and roasting processes of coffee beans. Notable examples include benzo(a)pyrene and anthracene, both of which were detected at trace levels in SCG.

PAH contamination during coffee processing often occurs due to smoke from fuel used in drying machines or from environmental pollution, such as air contamination near roads [38]. While specific regulatory limits for PAHs in coffee beans are not explicitly stated, the European Union has established limits for PAHs in certain foods under Commission Regulation (EU) 2023/915 [37]. Even though these limits may not directly apply to coffee beans, it remains crucial to minimize PAH contamination throughout the processing stages.

Data presented in Table 2 indicates that PAHs, including benzo(a)pyrene, were either below the limit of quantification or present in very low concentrations, suggesting that SCGs remain within safe levels for further use. Nonetheless, careful monitoring and control of drying and roasting practices are essential to prevent potential contamination and ensure the safety of SCGs, especially if repurposed for food or biofuel applications.

### 2.2. Protein Extraction and Precipitation

Table 3 presents the results of protein extraction and the retention of total polyphenols and antioxidant capacity across three extraction methods: mechanical stirring, ultrasound-assisted extraction, and CO_2_-assisted extraction, as well as the remaining spent coffee grounds (SCGs) after each method.

The highest protein yield was obtained using CO_2_-assisted extraction (34.24%), followed by mechanical stirring (31.46%) and ultrasound-assisted extraction (28.51%). The higher yield in CO_2_ extraction could be attributable to its high-pressure environment, which may enhance protein solubilization more effectively than the other methods. The ultrasound-assisted extraction showed the lowest protein yield, which might be due to the limitations of ultrasonic cavitation in extracting larger proteins from coffee grounds. In the SCG residue, the protein content was lowest after CO_2_ extraction (4.08%), indicating more efficient protein removal with this method. In contrast, ultrasound-treated SCGs retained slightly more protein (5.44%), suggesting incomplete extraction compared with the other methods. The ANOVA results showed that the differences in protein content between the methods were statistically significant (*p* < 0.05), particularly highlighting the superior protein extraction efficiency of the CO_2_-assisted method compared with ultrasound and stirring. When comparing the protein and bioactive compound content of the coffee extracts after different extraction methods (Table 3) to the initial values of spent coffee grounds (SCGs) before extraction (Table 1), it becomes clear that the extraction processes significantly enhance the yield of target compounds.

For total polyphenols, all extraction methods resulted in high concentrations in the coffee extracts, with CO_2_ extraction showing the highest value (29,755 mg GAE/kg), followed closely by mechanical stirring (29,403 mg GAE/kg) and ultrasound (28,896 mg GAE/kg). The differences between the methods are minimal, with standard deviations overlapping, indicating that all three methods are highly effective at preserving polyphenolic compounds. In the SCGs, the remaining polyphenols were relatively low, ranging from 488 mg GAE/kg after stirring to 509 mg GAE/kg after CO_2_ extraction. This indicates that a significant portion of polyphenols was successfully extracted from the coffee grounds across all methods. CO_2_ extraction left the least polyphenol residue in the SCGs, again reinforcing its efficiency.

The antioxidant capacity followed a similar trend to total polyphenols, with CO_2_ extraction showing the highest value (70.11 mg vit C/g), followed by stirring (69.43 mg vit C/g) and ultrasound (68.09 mg vit C/g). These values suggest that the polyphenolic compounds extracted are likely contributing to the antioxidant capacity of the extracts. The SCG residues retained minimal antioxidant activity, with values ranging from 1.14 mg vit C/g (stirring) to 1.20 mg vit C/g (CO_2_ extraction). This further confirms that the majority of the antioxidant compounds were successfully removed during extraction.

For total polyphenol content and antioxidant capacity, although there were slight differences in the mean values between the methods, the ANOVA indicated that these differences were not statistically significant (*p* > 0.05). This suggests that, despite CO_2_ extraction showing slightly higher values, all three extraction methods are comparably effective at preserving polyphenols and antioxidant compounds.

The results are similar for protein content to the ones found by Samsalee et al. (29.90–33.95%) but are much lower for total polyphenols (139.29–344.82 mg GAE/g SCG protein) and consequently of antioxidant capacity (591.63–976.07 mM Trolox eq/g SCG protein) [39]. Bhattarai, who also employed an alkaline extraction method, reported similar protein concentrations in spent coffee grounds, around 30% [40]

Data presented in Table 3 includes standard deviations, which indicate the variability between replicates for each extraction method. Notably, the CO_2_-assisted extraction method shows larger standard deviations in the protein content (±3.47%) compared with the other methods, suggesting some variability in performance. However, for total polyphenols and antioxidant capacity, the standard deviations are relatively small, indicating consistent extraction outcomes across methods.

CO_2_-assisted extraction emerges as the most efficient method for protein extraction from spent coffee grounds, offering the highest protein yield while also maintaining high levels of polyphenols and antioxidant activity. Mechanical stirring, though slightly less effective for protein extraction, remains highly efficient for bioactive compound retention. Ultrasound-assisted extraction, while more environmentally friendly, appears to be slightly less effective overall in comparison to the other methods.

### 2.3. Amino Acid Profile

**Figure 1:**
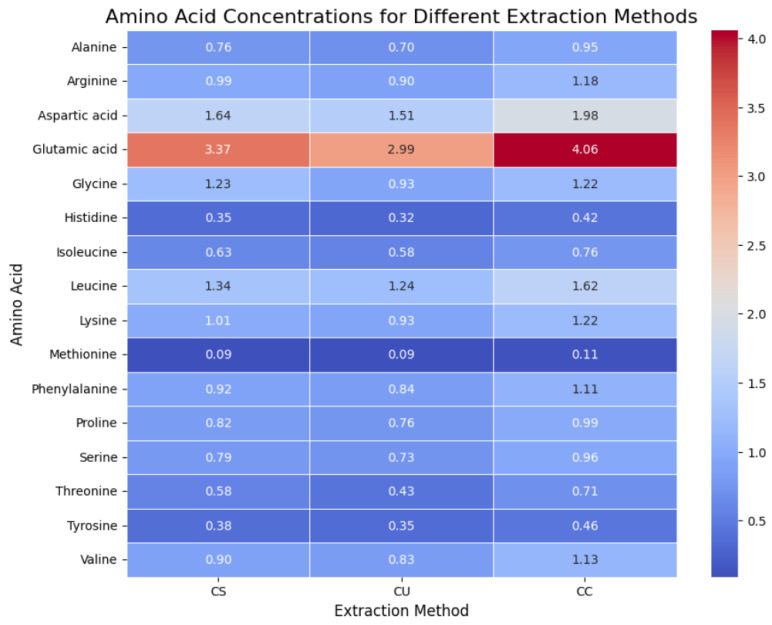
Amino acid profile in the 3 extracts (Coffee extract stirring (CS), Coffee extract ultrasound (CU), Coffee extract CO_2_ (CC)) in g/100g DW.

Across most amino acids, CO_2_-assisted extraction resulted in higher concentrations compared with mechanical stirring and ultrasound-assisted extraction (Figure 1). For instance, Glutamic acid is significantly higher in the CO_2_ extraction (4.06 g/100 g) compared with both stirring (2.99 g/100 g) and ultrasound extraction (3.37 g/100 g). This could be due to the high-pressure conditions used in CO_2_ extraction, which may more effectively solubilize and isolate proteins, including amino acids, from the matrix.

Other amino acids, such as leucine and isoleucine, both essential amino acids, also showed higher concentrations in the CO_2_ extraction method, suggesting that this technique is particularly effective for retaining essential amino acids in the protein extract. Ultrasound extraction generally results in amino acid concentrations higher than mechanical stirring but lower than CO_2_ extraction. For example, arginine concentrations in the ultrasound extract are 0.98 g/100 g, slightly higher than stirring (0.9 g/100 g) but lower than CO_2_ extraction (1.18 g/100 g). This method relies on ultrasonic cavitation, which helps to disrupt the SCG matrix, facilitating protein release but potentially less efficiently than CO_2_ extraction. Ultrasound extraction still performs well for some amino acids, such as glycine (1.23 g/100 g) and serine (0.79 g/100 g), indicating it can be an effective method for specific amino acids.

CO_2_-assisted extraction likely performs better because of the high pressure and lower temperature, which may protect proteins and amino acids from thermal degradation while also facilitating efficient extraction. It is particularly effective for amino acids that are more hydrophobic or larger, such as leucine and glutamic acid, which are key contributors to protein functionality. Ultrasound-assisted extraction provides a balance between efficiency and cost-effectiveness, offering relatively high amino acid yields without requiring the complex setup of CO_2_ extraction. It may be more suitable for applications where moderate amino acid extraction is sufficient.

Contrary to the expected trend, mechanical stirring demonstrated higher yields of most amino acids compared with ultrasound-assisted extraction. For example, alanine (0.76 g/100 g), arginine (0.98 g/100 g), and glycine (1.23 g/100 g) are present at higher concentrations in mechanical stirring than in ultrasound extraction. This suggests that, despite being a simpler and less intensive method, mechanical stirring may be more effective at releasing certain amino acids. The continuous agitation and controlled temperature in mechanical stirring could allow for better solubilization and extraction of proteins from the SCG matrix.

The statistical analysis and data comparison show that mechanical stirring is more effective than ultrasound-assisted extraction for the recovery of most amino acids from spent coffee grounds. However, CO_2_-assisted extraction remains the most effective method overall, yielding the highest concentrations of essential and non-essential amino acids. These findings suggest that mechanical stirring could be a cost-effective alternative to ultrasound-assisted extraction, while CO_2_-assisted extraction is the optimal method when maximizing amino acid recovery is a priority.

### 2.4. Mycelium-Based Packaging Material and Renewable Energy Pellet

Given that a substantial quantity of solid SCGs remain after protein extraction, it was important to explore its potential in other applications, such as mycelium-based packaging. To create the mycelium-based packaging, all SCGs produced from the extraction process were combined in equal parts. One key factor in determining the suitability of materials for such applications is their apparent density, which influences the structural integrity and material properties of the final product.

Table 4 provides the apparent density of different substrate materials used for mycelium-based packaging. Spent coffee grounds exhibited the highest apparent density, suggesting that SCGs offer a compact and relatively dense substrate, which could contribute to the strength and rigidity of mycelium-based composites.

ANOVA was performed to evaluate the significance of the differences in apparent density among the substrate materials. The analysis revealed significant differences (*p* < 0.05) between SCGs and lighter materials, such as coffee chaff, confirming that the higher density of SCGs makes them more suitable for applications requiring enhanced structural integrity. In contrast, the density values for sawdust and waste cereal mixtures were not significantly different from each other, indicating they could be interchangeable or blended based on the desired material properties. The substantial density difference between SCGs and coffee chaff also suggests that combining these materials could offer a balanced solution—where coffee chaff provides lightweight characteristics, and SCGs contribute strength and rigidity to the mycelium-based packaging.

**Figure 2:**
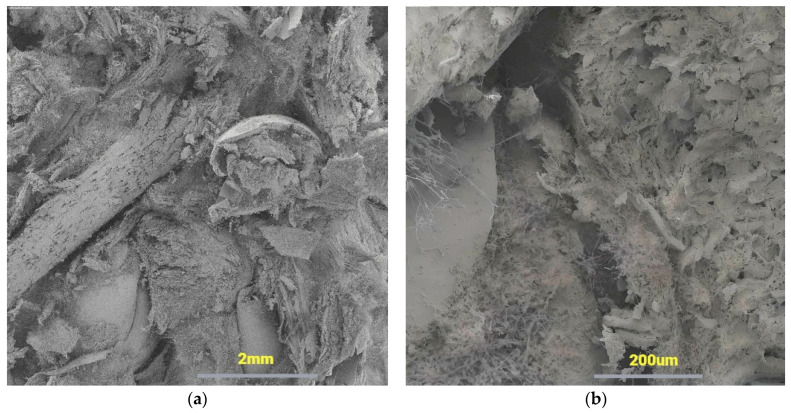
Sample image at (**a**) 16× and (**b**) 191× magnification.

SEM images at different magnifications of the mycelium base material are presented in Figure 2. The sample displays a compact construct at low magnification (16×), while air holes can be observed at higher magnification (191×). The presence of air holes in the sample can influence its compressive strength.

**Figure 3:**
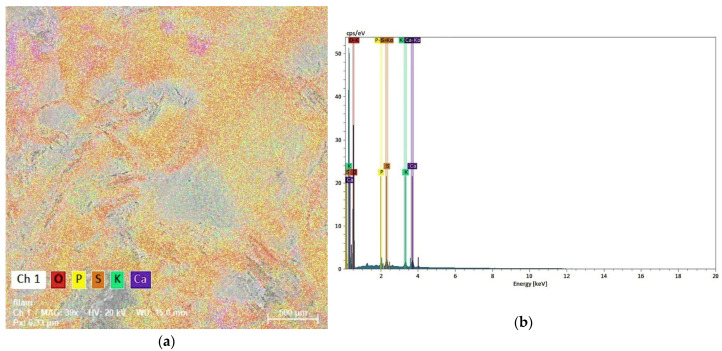
(**a**) Elemental map and (**b**) EDX spectrum of the sample.

An EDX measurement was performed on the sample. The component elements are presented in “false” colors (Figure 3a). The spectrum (Figure 3b) shows the presence of P, S, K, and Ca as major elements in the sample. In earlier studies, P, K, and Ca were identified as some of the major components of coffee, one of the major elements from the sample [41].

**Figure 4:**
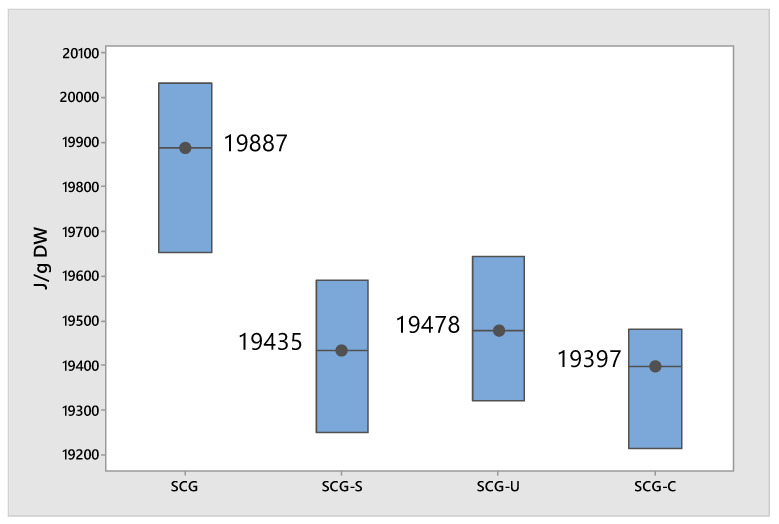
Energy content of SCGs before and after extraction. The extraction methods used are SCG-S (mechanical stirring), SCG-U (ultrasound-assisted), and SCG-C (CO_2_-assisted).

The energy values for SCGs and the SCGs after extraction (SCG-S (mechanical stirring), SCG-U (ultrasound-assisted), and SCG-C (CO_2_-assisted)) show slight variations (Figure 4). The SCGs before extraction have an energy value of 19,887 J/g DW, while the values after extraction range from 19,397 J/g DW to 19,578 J/g DW. The differences between these values are minor, as confirmed by ANOVA (*p* > 0.05), indicating no statistically significant differences between the different extraction methods. However, when comparing the initial SCGs to the SCGs after extraction, the ANOVA analysis revealed a statistically significant difference (*p* < 0.05). The extraction process does have a measurable effect on the energy content of SCGs. However, while this effect is statistically significant when comparing the SCGs before and after extraction, the variation in energy content between the different extraction methods (mechanical stirring, ultrasound-assisted, and CO_2_-assisted) is not significant. This indicates that although the extraction process alters the energy content overall, the specific method used does not lead to substantial differences in the resulting energy values. While the energy content decreases slightly after extraction, the impact of the extraction method on the energy value is minimal, and the energy content remains relatively high, indicating that SCGs could still be a useful source of energy even after protein extraction.

**Figure 5:**
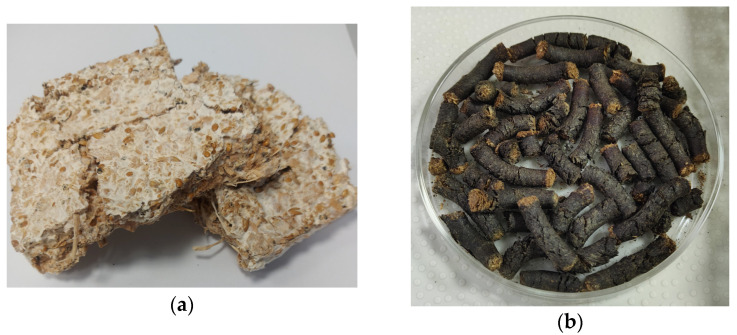
(**a**) Mycelium-based packaging material after compression test and (**b**) Coffee waste and pine wood pellets.

Figure 5a depicts the mycelium-based packaging material after undergoing a compression test. The material appears compacted but retains some structural integrity, as evidenced by the visible interconnected fibers. This highlights its ability to withstand compressive forces while maintaining form, which is consistent with the high compression resistance observed in these data (3.354 MPa) (Table 5). The mycelium network contributes to the material’s robustness, making it suitable for packaging applications that require durable and eco-friendly alternatives to conventional materials. Optimizing growth conditions such as temperature, humidity, and incubation time may result in a more uniform mycelial network, improving the structural consistency and reducing variability in the material’s mechanical properties (currently indicated by a standard deviation of ±0.97 MPa). Fine-tuning these parameters could also decrease production time without compromising strength.

Figure 5b shows the coffee waste and pine wood pellets. These pellets are less compact and more fragmented, which correlates with the significantly lower compression resistance observed in these data (0.834 MPa).

Nosek et al. reported that 100% SCGs had the highest lower calorific value (LCV) at 21.08 MJ/kg, while the mixture of SCGs with sawdust (30/70) yielded 20 MJ/kg, which is similar to the results in our study, were the energy values for SCGs before extraction were measured at 19,887 J/g DW (equivalent to 19.887 MJ/kg), and after extraction, they ranged from 19,397 J/g DW to 19,578 J/g DW [42].

Adjusting the moisture content during pellet formation could improve the material’s compressive strength. Pellets with too high or too low moisture content might not form properly or hold together, leading to lower mechanical strength.

Optimizing the composition and production parameters for both the mycelium-based packaging material and coffee waste and pine wood pellets will enhance their mechanical properties and broaden their potential applications. For the mycelium-based packaging, the focus would be on maximizing strength and durability, while for the pellets, improving cohesion and energy efficiency would be key for their use as a sustainable fuel.

## 3. Materials and Methods

### 3.1. Samples Collection

The coffee waste samples (spent coffee grounds and coffee chaff) were collected from May to August 2024 from the MERON Coffee shop chain from Cluj-Napoca, Romania. All SCG samples were derived from the same type of coffee (Arabica, Kyoto, Japan). In total, a number of 8 sampling events were performed for SCGs and the sample quantity varied from 2.5–4.7 kg. Only one sampling event was conducted for coffee chaff, which was collected during the roasting process at MERON Coffee using a cyclone system to separate the chaff from the roasted beans. Collected samples were manually cleaned of any impurities, dried (UFE 400 oven from Memmert, Büchenbach, Germany) until the dry matter reached 95%, and stored in a cold, dark place until processing. An analysis for characterization of the SCGs was performed for each sampling campaign.

### 3.2. Reagents and Materials

All solvents were HPLC grade from VWR (Darmstadt, Germany), with ultra-pure wa-ter obtained using the ULTRACLEAR UV UF EVOQUA Purification system (Pittsburgh, PA, USA), ACW Kit from Analitk Jena (Jena, Germania), immunoaffinity columns for aflatoxin determination 3 mL AFLASTAR^®^ IA, BIOPURE Micotoxin Mix 1 (Aflatoxins), in acetonitril, 5 mL, from Romer Labs (Butzbach, Germany), OPA reagent, 10 mg/mL each of o-phthalaldehyde and 3-mercaptopropionic acid in 0.4 M borate buffer, 6 × 1 mL from Agilent (Santa Clara, CA, USA), Sodium hydroxide CS reagent, ≥97.0%, pellets, Folin-Ciocalteu′s phenol reagent, PAH Calibration Mix certified reference material, 10 μg/mL each component in acetonitrile, Gallic acid monohydrate ACS reagent, ≥98.0%, L-Ascorbic acid BioXtra, ≥99.0%, crystalline, Amino Acid Standard from Sigma-Aldrich (Saint Louis, MO, USA).

### 3.3. Protein Extraction

Protein extraction from spent coffee grounds was performed using three distinct methods: mechanical stirring, ultrasound-assisted extraction, and CO_2_-assisted extraction. Each method was selected to optimize protein yield while minimizing the environmental impact of the process.

For each extraction method, 100 g of spent coffee grounds was mixed with 500 mL distilled water, and the pH was brought to 10.5 using a 10% NaOH water solution.

#### 3.3.1. Mechanical Stirring

The extraction was performed for 20 min at 400 rpm (OS20-Pro mechanical stirring from DLAB Johor, Malaysia) and 40 °C (RET bacis, Magnetic Stirrer HotPlate from IKA, Staufen, Germany).

#### 3.3.2. Ultrasound-Assisted Extraction

The extraction was performed in an ultrasonic bath (SONOREX, Bandelin, RK 103H, Berlin, Germany) at 59 kHz for 20 min, and the temperature was set at 40 °C.

#### 3.3.3. CO_2_-Assisted Extraction

High-pressure CO_2_ extraction was performed using a Parr Instruments 1-L benchtop reactor with a 4875 Power Controller (Parr Instrument Company, Moline, IL, USA). The 20-min extraction was performed at a pressure between 55.5 and 57.8 bar, and the temperature was between 20.7 and 22.3 °C at 11,000 rpm.

### 3.4. Protein Precipitation

After decantation, the liquid portion was transferred to a new beaker, and 10 g of ascorbic acid was added, reducing the pH to 3.5–4 to facilitate protein precipitation. The precipitated proteins were then separated by centrifugation (Megafuge 16, Thermo Fisher Scientific, Waltham, MA, USA) and subsequently lyophilized (BK-FD18T, Biobase, Jinan, China). The lyophilized proteins were stored at 4 °C for further use.

### 3.5. Mycelium-Based Packaging Material

The substrate composition significantly impacts the behavior of mycelium-based composites. In our study, we developed a balanced blend through a combination of insights from previous studies and our own optimization tests. The blend consisted of:

∘ Spent coffee grounds after protein extractions (a mixture of SCGs from mechanical stirring, ultrasound-assisted, and CO_2_-assisted methods)—30%;
∘ Coffee chaff—10%;
∘ Sawdust—40%;
∘ Waste cereal mixture—5%;
∘ Ganoderma lucidum mycelium—15%.

#### 3.5.1. Substrate Preparation of Mycelium-Based Composites

The raw materials had been ground to acquire a uniform distribution of grain sizes (2.4–4 mm), and the substrate had been supplied with a moisture content of 60–75% before being sterilized at 121 °C for 30 min to ensure material composition decontamination.

#### 3.5.2. Inoculation

Spent coffee grounds, sawdust, waste cereal combination, and coffee chaff were inoculated with 15% spores of the fungal species G. lucidum. A second layer of straw, enriched with 20–35% moisture, was spread out in 100 × 100 mm molds. The raw materials mixture was then compressed to a thickness of 50 mm and covered with a sterile, biodegradable foil. This layered approach provides a conducive environment for mycelium growth and shields the substrate during the critical early stages of mycelial development; moreover, controlled pressure ensures proper substrate density, allowing mycelium to colonize effectively.

#### 3.5.3. Incubation

Mycelium incubation is a dynamic phase where temperature regulation and substrate interactions profoundly impact growth. The optimal temperature for mycelium growth during this stage is maintained at 24.3 ± 3.5 °C in darkness to mimic natural subterranean conditions.

During the incubation process (INE 400 Incubator, Memmert, Büchenbach, Germany), the mycelium extended its hyphal network throughout the substrate, utilizing the substrate as both a structural support and a nutrient source. To optimize aeration and ensure uniform mycelial development, the incubation period was divided into three distinct phases. In the initial phase, the growth of mycelium was directed horizontally across the substrate. This was followed by a vertical growth phase, where the focus shifted to upward expansion. In the final phase, mycelium was allowed to proliferate freely without any structural constraints. To address moisture loss, particularly during vertical growth, the samples were rotated 180 degrees to counteract moisture migration caused by gravity. This rotation was critical in maintaining consistent moisture levels throughout the substrate, particularly in the upper layers, preventing desiccation. Continuous monitoring of the mycelium growth was performed throughout the incubation period to ensure optimal development and the formation of a robust mycelial network.

For the inoculated samples of *G. lucidum*, the incubation period lasted 10 days. During the first growth phase, an experimental sample was observed after 40 h of incubation, with the upper surface exposed to air.

#### 3.5.4. Drying Method

The drying process of the samples was performed in a laboratory oven (UFE 400 oven from Memmert, Büchenbach, Germany) to prevent mycelial growth and remove excess moisture. The drying time is 4 to 6 h, and the temperature range is 80 °C to 90 °C. When the mycelium growth stops by thermal treatment, its filaments are no longer supported by the internal hydrostatic pressure, and for this reason, they appear to be flattened. The samples were prepared in heat-resistant molds (100 × 100 × 50 mm) with a formulation of 90% matrix and 10% fungal spores in the composite. The initial thickness at the beginning was 50 mm, but after the drying process, the final thickness of the experimental sample reached 35 mm due to the removal of excess moisture.

### 3.6. Renewable Energy Pellets

To produce renewable energy pellets, a mixture of 1 part spent coffee grounds (after protein extraction) and 2 parts pine wood sawdust and chips were used. The materials were thoroughly mixed and fed into a pellet mill (KL-120, Uniteh Pro, Botosani, Romania) equipped with a 3 kW motor capable of processing up to 100 kg/h equipped with a sieve of 6 mm size. The machine compresses the mixture into pellets through high pressure and temperature, creating dense, durable energy pellets suitable for combustion.

### 3.7. Pycnometer Method for Measurement of Apparent Density for Substrate Materials

The apparent density of substrate materials, including coffee grounds waste, coffee chaff, sawdust, and waste cereal mixture, was measured using a pycnometer (Pycnomatic ATC, Thermo Fisher Scientific, USA). The pycnometer was filled with each substrate material and weighed accurately to determine the mass of the sample. The apparent density was calculated by dividing the mass of the substrate by the known volume of the pycnometer (100 mL). All measurements were performed in triplicate to ensure accuracy, and the results were averaged to provide a consistent comparison across the different substrate materials.

### 3.8. Ash Content Analysis

Ash content analysis was performed according to the ISO 2171:2023 standard [43]. Approximately 5 g of sample was placed in a crucible and incinerated in a muffle furnace at 550 °C until complete combustion. The samples were cooled in a desiccator and weighed to determine the ash content. The results were expressed as a percentage of the initial sample weight.

### 3.9. Total Lipid Content (Soxhlet Extraction Method)

Total lipid content analysis was performed using a simplified Soxhlet extraction method [44]. Approximately 5 g of dried, homogenized sample was placed in an extraction thimble and extracted with petroleum ether in a Soxhlet apparatus for 4–6 h. After extraction, the solvent was evaporated, and the lipid residue was dried at 105 °C to constant weight. The lipid content was determined gravimetrically and expressed as a percentage of the initial sample weight.

### 3.10. Total Protein Content (Kjeldahl Method)

Total protein content analysis was performed according to the ISO 937:2023 standard [45]. Approximately 1–2 g of homogenized sample was digested with 15 mL of concentrated sulfuric acid and a catalyst in a Kjeldahl digestion unit at 350–400 °C until the solution became clear. After digestion, the sample was neutralized with 40% sodium hydroxide solution and distilled using a Kjeldahl distillation unit. The ammonia was collected in 4% boric acid and titrated with 0.1 N hydrochloric acid using bromocresol green/methyl red indicators. The nitrogen content was calculated and converted to protein content using a factor of 6.25. The results were expressed as a percentage of the initial sample weight.

### 3.11. Folin Ciocalteu Method for the Determination of the Total Polyphenolic

A 0.5 g portion of the homogenized sample was extracted with 10 mL of methanol (MeOH) using a vortex mixer (Vortex 2, IKA, Staufen, Germany) for 2 min at a speed of 2500 rpm. The mixture was then centrifuged at 11,000 rpm for 2 min, and the supernatant was filtered through a 0.45 µm cellulose membrane filter.

An adapted method from Bobková et al. was employed [46]. In a 15 mL centrifuge tube, 5 mL of distilled water, 1.5 mL of 10% sodium carbonate solution, 0.5 mL of the sample, and 0.5 mL of Folin–Ciocalteu reagent were combined. The samples were left in the dark at room temperature for 45 min, after which absorbance was measured at 765 nm using a spectrophotometer (Lambda 25, Perkin Elmer, Waltham, MA, USA). Results were expressed in gallic acid equivalents.

### 3.12. Total Antioxidant Capacity

Following the same methanol extraction procedure as used in the Folin–Ciocalteu method, the samples were directly injected into the PHOTOCHEM analyzer (Analytik Jena, Jena, Germany). The antioxidant capacity was measured using the ACW kit and expressed in terms of equivalent vitamin C.

### 3.13. Aflatoxin Analysis

The analysis followed the EN ISO 16050:2011 standard [47]. Briefly, a 25 g sample was extracted using a methanol-water solvent mixture (70:30 *v*/*v*) with 5 g of sodium chloride (NaCl) added to enhance extraction efficiency. The extract was filtered, diluted with water, and purified using an immunoaffinity column containing antibodies specific for aflatoxins B1, B2, G1, and G2 (3 mL AFLASTAR^®^ IA, Romer Labs, Butzbach, Germany). The methanol eluate was collected and injected into the HPLC system.

The HPLC analysis was performed using a Perkin Elmer 200 Series system with a fluorescence detector (Waltham, MA, USA) and post-column derivatization (Derivatization Unit, Romer Labs, Butzbach, Germany). Separation was achieved on a Tracer Excel 120 ODS-B column (5 µm, 15 cm × 0.46 cm; Teknokroma Analítica, Barcelona, Spain) with isocratic flow at 0.7 mL/min. The injection volume was 40 µL, and detection was carried out at an excitation wavelength of 360 nm and emission at 440 nm. The column compartment was maintained at 35 °C.

### 3.14. PAHs Analysis

The method was adapted from a previously developed approach for analyzing various food samples using high-performance liquid chromatography (HPLC) with fluorimetric detection following sonication extraction, as described by Nieva-Cano et al. [48]. A Perkin Elmer 200 Series High-Performance Liquid Chromatograph (HPLC) with a fluorescence detector (FLD) was employed. Separation of 15 polycyclic aromatic hydrocarbons (PAHs), including naphthalene, acenaphthene, fluorene, phenanthrene, anthracene, fluoranthene, pyrene, benzo(a)anthracene, chrysene, benzo(b)fluoranthene, benzo(k)fluoranthene, benzo(a)pyrene, dibenzo(a,h)anthracene, benzo(g,h,i)perylene, and indeno(1,2,3-cd)pyrene, was performed on an Inertsil ODS-P column (5 µm, 4.6 × 150 mm) maintained at 24 °C. The injection volume was 50 µL, and the mobile phase consisted of a gradient of water and acetonitrile. A time-programmed FLD detector was used for PAH detection, with results expressed in ng/g.

### 3.15. Amino Acid Profile

The method, which is a modified version of the protocol from Synaridou et al. (2021), involved hydrolyzing 5 g of homogenized sample with 20 mL of 4 M HCl at 95 °C for 24 h [49]. After filtration through filter paper, the samples were neutralized with 15 mL of a 10% KOH solution. The neutralized samples were then filtered again using a 0.45 µm filter and injected into the HPLC system. Pre-column derivatization was performed with ortho-phthalaldehyde (OPA) in a 1:2 ratio controlled through the automatic sample injection system. The analysis was conducted using a Vanquish UHPLC system equipped with a fluorescence detector (Thermo Fisher Scientific, Waltham, MA, USA). The excitation and emission wavelengths were set at 340 nm and 450 nm, respectively. A mobile phase of ultrapure water and acetonitrile was used in a gradient system with a flow rate of 0.800 mL/min. Chromatographic separation was achieved on a Hypersil Gold column (150 × 4.6 mm, 5 µm particle size), and the injection volume was 1 µL. The column thermostat was maintained at 25 °C.

### 3.16. Determination of Calorific Value

The higher heating value (HCV) was determined using a 6200 isoperibol calorimeter (Parr Instrument, Moline, IL, USA) calibrated by burning certified benzoic acid. The weighed sample containing 0.4 g biomass and 0.6 g benzoic acid was placed in the sampler holder of the bomb. The bomb was assembled, filled with oxygen for 30 s at a pressure of 400 psi, and placed in the calorimeter. The sample was combusted under controlled conditions for 15 min (temperature was recorded during combustion). 

### 3.17. SEM Analysis

The sample (~1.5 × 0.5 mm size) was coated with a 15 nm gold layer using Leica EM ACE200 (LEICA, Wetzlar, Germany) equipment. The surface of the sample was analyzed with a Scanning electron microscope (VEGA3 SBU, Tescan, Brno-Kohoutovice, Czech Republic) with an energy dispersive X-ray spectroscope (Quantax EDS, Bruker, Karlsruhe, Germany) SEM/EDX, HV = 5 kV, at room temperature.

### 3.18. Mechanical Properties Analysis

The mechanical properties of pallets and the mycelium base material were performed according to ASTM D695 on a Compression Testing Machine (Utest Material Testing Equipment, Ankara, Turkey) [50]. The test involved applying a compressive load of 1% at a uniform rate of 1.3 mm/min until failure (failure threshold was set to 40 kN) or a predetermined deformation was reached. This allowed for the accurate measurement of compressive strength, strain, and modulus, ensuring consistent and reliable data for evaluating the material’s performance under compressive stress.

### 3.19. Statistical Evaluation

The results were expressed as the mean ± standard deviation, with each sample analyzed in triplicate. A one-way analysis of variance (ANOVA) was performed using Minitab for Windows version 17.0 (Minitab, LLC, State College, PA, USA). Graphics were created using Python with pandas, seaborn, and matplotlib.pyplot libraries and Minitab 17.0.

## 4. Conclusions

This study demonstrates the promising potential of spent coffee grounds (SCGs) as a sustainable resource. CO_2_-assisted extraction emerged as the most effective method for protein recovery, yielding 34.24%, higher than mechanical stirring and ultrasound-assisted methods. SCGs also showed excellent applicability in mycelium-based packaging, providing a dense, structurally sound material with a compression resistance of 3.354 MPa, indicating its viability for eco-friendly packaging solutions. Additionally, SCG-based pellets retained high calorific values, confirming their potential as a renewable energy source. Future research should prioritize optimizing protein extraction techniques and exploring the potential of SCG-derived proteins for aquaculture and human consumption. Enhancing the mechanical properties of SCG-based materials will also expand their commercial applications, contributing to a circular economy by reducing waste and promoting resource efficiency.

## Figures and Tables

**Table 1:** Composition of Spent Coffee Grounds (SCGs).

Determination	Average ^1^	Units
Protein	15.77 ± 0.58	% DW
Lipids	15.25 ± 0.24	% DW
Ash	2.89 ± 0.02	% DW
Total polyphenol	1113 ± 368	mg GAE/kg
Antioxidant capacity	2.60 ± 0.02	mg vit C/g
Aflatoxin B1	0.25 ± 0.15	µg/kg
Aflatoxin B2	<LQ ^2^	µg/kg
Aflatoxin G1	<LQ ^3^	µg/kg
Aflatoxin G2	<LQ ^2^	µg/kg

^1^ All values are expressed as the mean ± standard deviation (SD) for each determination; ^2^ LQ ≥ 0.04 µg/kg; ^3^ LQ ≥ 0.15 µg/kg.

**Table 2:** PAHs concentration in SCGs (µg/kg).

Determination	Average ^1^
Naphthalene	0.0021 ± 0.014
Acenaphthene	<LQ ^2^
Fluorene	<LQ
Phenanthrene	0.0066 ± 0.0032
Anthracene	0.0003 ± 0.00007
Fluoranthene	0.0031 ± 0.018
Pyrene	0.0013 ± 0.0009
Benz[a]anthracene	0.0004 ± 0.0005
Chrysene	0.0002 ± 0.0004
Benzo[b]fluoranthene	<LQ
Benzo[k]fluoranthene	<LQ
Benzo[a]pyrene	<LQ
Dibenz[a,h]anthracene	<LQ
Benzo[ghi]perylene	0.0001 ± 0.0003
Indeno[1,2,3-cd]pyrene	0.0001 ± 0.0002
TOTAL PAH	0.0141 ± 0.0052

^1^ All values are expressed as the mean ± standard deviation (SD) for each determination; ^2^ LQ ≥ 0.0001 µg/kg.

**Table 3:** Evaluation of protein extraction methods and bioactive compounds retention in spent coffee grounds using stirring, ultrasound, and CO_2_-assisted techniques ^1^.

Determination	Protein (%)	Total Polyphenol (mg GAE/kg)	Antioxidant Capacity (mg vit C/g)
Coffee extract stirring (CS)	31.46 ± 0.52	29,403 ± 170	69.43 ± 1.94
Coffee extract ultrasound (CU)	28.51 ± 1.09	28,896 ± 133	68.09 ± 3.67
Coffee extract CO_2_ (CC)	34.24 ± 3.47	29,755 ± 186	70.11 ± 3.97
SCGs after stirring extraction (SCG-S)	4.41 ± 0.13	488 ± 4.38	1.14 ± 0.02
SCGs after ultrasound extraction (SCG-U)	5.44 ± 0.21	50 ± 6.18	1.18 ± 0.02
SCGs after CO_2_ extraction (SCG-C)	4.08 ± 0.35	509 ± 7.78	1.20 ± 0.01

^1^ All values are expressed as the mean ± standard deviation (SD) for each determination.

**Table 4:** Apparent density of the materials used for mycelium-based packaging (g/cm^3^).

Substrate Material	Average ^1^
SCGs after extractions	0.551 ± 0.04
Coffee chaff	0.079 ± 0.02
Sawdust	0.513 ± 0.05
Waste cereal mixture	0.505 ± 0.07

^1^ All values are expressed as the mean ± standard deviation (SD) for each determination.

**Table 5:** Compression resistance of the mycelium-based packaging material and coffee waste and pine wood pellets (MPa).

Substrate Material	Average ^1^
Mycelium-Based Packaging Material	3.354 ± 0.97
Coffee waste and pine wood sawdust pellets	0.834 ± 0.12

^1^ All values are expressed as the mean ± standard deviation (SD) for each determination.

## Data Availability

The data presented in this study are available upon request from the corresponding author. The data are not publicly available due to privacy restrictions.
